# Prevalence of vancomycin-resistant enterococcus in Africa in one health approach: a systematic review and meta-analysis

**DOI:** 10.1038/s41598-020-77696-6

**Published:** 2020-11-25

**Authors:** Tsegaye Alemayehu, Mengistu Hailemariam

**Affiliations:** School of Medical Laboratory Sciences, Hawassa University College of Medicine and Health Sciences, P.O. Box 1560, Hawassa, Sidama Ethiopia

**Keywords:** Microbiology, Environmental sciences, Diseases

## Abstract

Vancomycin-resistant enterococci are a global challenge currently as reported by the World Health Organization. It is also important to recognize that combating antimicrobial resistance needs to recognize the interconnections between people, animals, plants and their shared environment in creating public health, the so-called One Health approach. Although the presence of VRE has been described in many regions of the world, there is a lack of comprehensive data indicating their prevalence of in Africa. Therefore, this study aimed to aggregate the result of studies describing VRE reported across multiple regions in Africa. A literature search was conducted on PubMed, Google scholar, and Hinari with the term “Vancomycin resistance enterococcus in Africa” on August 1–3, 2019. All available articles were downloaded to “Endnote version 7.1” then to Microsoft Word 2013. Articles determined to meet our criteria for the review was extracted to Microsoft Excel 2013. Those articles that reported the prevalence of vancomycin resistance *Enterococcus* obtained from all sample types and published from 2010 to 2019 in the English language were included for the review. A meta-analysis was conducted with OpenMetaAnalyst version R.3.1.0 software. The effect size was determined using a binary random effect model and statically significant considered when *p* < 0.05. Heterogeneity determined with the inconsistency index. A leave one out analysis used to perform the sensitivity analysis. There were 151 articles identified from the database searches; of this, 36 articles included after extensive review with two independent authors. Out of 4073 samples collected, 1488 isolates identified with an overall pooled prevalence of VRE 26.8% (95% CI; 10.7–43.0%) in Africa with a one-health perspective. The analysis showed that considerable heterogeneity among the studies (I^2^ = 99.97%; *p* < 0.001). Subgroup analysis in-country, African region, laboratory method, year of publication, and sample source showed that a high prevalence was identified from South Africa (74.8%), South African regions (74.8%), PCR (959.2%), 2010–2015 years (30.3%) and environmental (52.2%), respectively. This meta-analysis indicates that there was a high-pooled prevalence of vancomycin-resistant enterococci in African. A lot should be done to prevent and control the transmission of vancomycin resistance enterococci to a human being from the environment in the continent.

## Introduction

Vancomycin-resistant enterococci are defined as members of the genus, *Enterococcus*, that possess either intrinsic or acquired resistance to the antibiotic vancomycin used to treat serious infections caused by these bacteria^1,2^. Intrinsic resistance occurs in isolates of *E. gallinarum* and *E. casseliflavus /E. flavescens*, which demonstrate an innate, low-level resistance to vancomycin. These enterococci rarely cause infections in humans or animals. In contrast, high-level vancomycin resistance, most commonly seen in *E. feacium* and *E. faecalis*, may be associated with serious, life-threatening infections. High-level vancomycin resistance has also been identified in *E. raffinosus*, *E. avium*, *E. durans*, and several other enterococci, however, these species are rarely associated with infections. A variety of transferable genetic elements designated *van*A, *van*B, *van*C, *van*D, and *van*E, may lead to resistance to vancomycin in enterococci, however, *vanA* and *vanB* are most common^3,4^.

VRE emerged as important nosocomial pathogens in the 1980s, and there is concern that they may be, or become, endemic in the non-hospital setting, both in human and animal reservoirs and in the general environment^5^. It advanced to an inoffensive colonizer of the gut of humans and animals, ranging from insects to reptiles, birds, and mammals. Whilst they are ubiquitous, they represent a minority population of the healthy human microbiome^6^. Presence in the environment is generally considered an indicator of human or animal faecal contamination of recreational or drinking water^7^.

The rise of VR *Enterococcus feacium* in the European Union has to lead to the sanction of avoparcin, an antibiotic that chemically related to vancomycin^8^. The USA never approved avoparcin for clinical use. In the years post-ban, VRE surveillance data of EU hospitals showed no obvious reduction in VRE rates. Because of very limited alternatives to vancomycin, VRE infections remain a serious clinical treatment challenge throughout the world. Surveillance studies showed zero rates of VRE in US livestock. Whole-genome sequencing data suggest that VRE might have evolved from ampicillin-resistant *E. feacium* from dogs^9^.

Some members of the genus *Enterococcus* are well-documented pathogens associated with serious clinical manifestations in humans, including bacteremia, infective endocarditis, intra-abdominal and pelvic infections, urinary tract infections, and, in rare cases, central nervous system infections^10–12^. Infection with VREs is associated with an increased mortality rate, illustrated by a 2.5-fold increase in mortality for patients suffering from VRE bacteremia^13^.

The One Health Commission defines One Health as “a collaborative, multisectoral, and trans-disciplinary approach—working at local, regional, national, and global levels—to achieve optimal health and well-being outcomes recognizing the interconnections between people, animals, plants and their shared environment.” All potentially constitute overlapping reservoirs of antimicrobial resistance^14^. Given the serious health threat, a common understanding of AMR, and of the need for a One Health approach to tackle it, are of fundamental importance^15,16^.

VRE is one of these multidrug resistances that need comprehensive data that indicates the pooled prevalence of VRE in Africa. Therefore, this study aimed to compile available data of VRE in Africa in a one-health perspective: a systematic review and meta-analysis.

## Methods

### Literature search strategy

**Figure 1:**
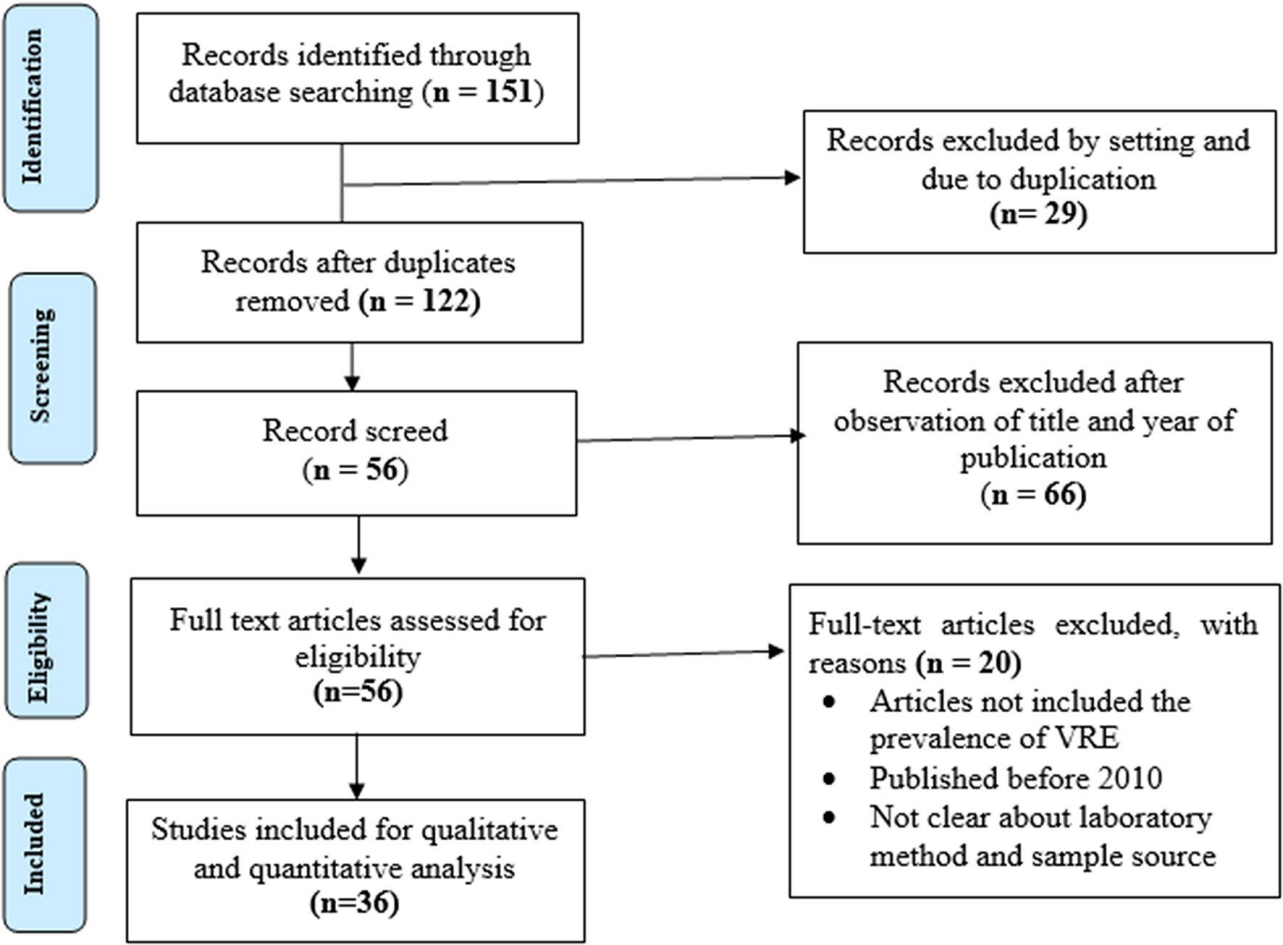
The PRISMA flow diagram for the selection of articles on the prevalence of vancomycin resistance enterococcus in Africa in a one-health approach: a systematic review and meta-analysis.

A literature search conducted on PubMed, Google scholar, and Hinari with the term “Vancomycin resistance enterococcus in Africa” on August 1–3, 2019. Citations of all available articles were exported to “Endnote version 7.1” then to Microsoft Word 2013. All the articles that met our inclusion criteria were included for systematic review and meta-analysis. There were 151 articles obtained from the databases. Of these, 29 articles were excluded based on setting and duplications, 66 were excluded because either title or year of publication was unacceptable. A total of 56 articles underwent full-text assessment. An additional 20 articles were excluded because they failed to report the prevalence of VRE, their year of publication was before 2010, or they lacked clearly described laboratory methods or unknown sample source. Finally, 36 articles were subjected to an extensive review by two independent authors. The article selection process was conducted according to the PRISMA protocol of 2015^17^ (Fig. 1).

### Eligibility criteria

The inclusion criteria for this review were articles published in the English language that reported the prevalence of VRE and were published from 2010 through 2019. All sample sources were considered. Specifically, publications excluded from this review were any of the following: published before 2010 or after 2019, published other than the English language, has no clear laboratory methods, had unknown sample types, or failed to include the prevalence of VRE.

### Data analysis

Microsoft Excel 2013 was used for data extraction and results were then exported to Microsoft Word plus 2013. Data was entered to OpenMetaAnalyst version R.3.1.0^18^ software for each study after copying each column from Excel to the software and a meta-analysis, subgroup meta-analysis and sensitivity analysis were conducted. The result was presented as a forest plot in the figure. The pooled prevalence of VRE at 95% CI was determined with a binary random-effect model by the DerSimonian–Laird method. The statistical significance was considered when *p* value < 0.05.

### Data quality

The quality of the study included in the review and meta-analysis evaluated with a 14 point scoring tool, an NIH quality assessment tool for observational and cross-sectional studies in which studies categorized as a good, fair, and poor quality based on the internal validity of each article^19^. Accordingly, nine (25%) articles were categorized as fair, eleven (30.6%) as poor, and sixteen (44.4%) articles as good quality (supplementary file).

### Heterogeneity and publication bias

The heterogeneity of the publication was determined with the measure of the inconsistency index (I^2^) and *p* value. The total variations in the articles were due to heterogeneity rather than by chance with a value of < 30%, 30–60%, 61–75%, and > 75% suggestive of low, moderate, substantial, and considerable heterogeneity, respectively^20^. Publication bias was not checked as the study is considerably heterogeneous as recommended by Hak et al.^21^, if the data is heterogeneous no need of conducting publication bias.

### Study features

Studies conducted in African countries that reported the prevalence of VRE and were published between 2010 and 2019 were considered. All sample types and laboratory methods employed were included for the review and meta-analysis. The following data types were extracted from each article and presented in Table 1: author name; year of publication; country of origin, source of sample (human, animal, environmental); laboratory method used (culture and polymerase chain reaction (PCR), PCR only, culture, number of different *Enterococcus* species isolated and the number of VRE isolated.

**Table 1:** Articles meeting inclusion criteria describing the prevalence of VRE in Africa in a one-health approach: a systematic review and meta-analysis.

Author, year	Country	Source of sample	One health segment	Lab. method	Enterococcus	VRE
Bouamama et al.^46^	Morocco	Flies and cockroaches	Animal	Culture	29	0
Djahmi et al.^56^	Algeria	Clinical specimen	Human	Culture and PCR	125	4
Ateba et al.^30^	South Africa	Groundwater	Environmental	PCR	179	166
Kateete et al.^50^	Uganda	Milkmen and cows mastitis	Animal and human	Culture	24	3
Moemen et al.^40^	Egypt	Clinical specimen	Human	Culture	52	12
Abebe et al.^23^	Ethiopia	Stool sample	Human	Culture	201	11
Katakweba et al.^54^	Tanzania	Buffalo, zebra, cattle and wildebeest faecal	Animal	Culture and PCR	120	10
Naouel et al.^45^	Tunisia	Faeces of birds	Animal	Culture and PCR	73	6
Anyanwu et al.^52^	Nigeria	Cattle rectal swab	Animal	Culture	75	5
Iweriebor et al.^32^	South Africa	Pig faeces	Animal	Culture and PCR	320	320
Hammad et al.^38^	Egypt	Milk cheese	Animal	Culture and PCR	120	6
Benson et al.^31^	South Africa	Hospital wastewater	Environmental	Culture and PCR	62	60
Abamecha et al.^22^	Ethiopia	Patients faeces	Human	Culture	142	7
Iweriebor et al.^33^	South Africa	Cattles	Animal	Culture and PCR	340	340
Dziri et al.^44^	Tunisia	Hospital env’t sample	Environmental	Culture	100	6
Ben Said et al.^42^	Tunisia	Vegetable, soil and water	Environmental	Culture	65	4
Nadjette et al.^57^	Algeria	Clinical specimen	Human	Culture	85	2
Hannaoui et al.^48^	Morocco	Faecal specimen	Human	Culture and PCR	100	21
Molale1 and Cornelis ^35^	South Africa	Surface water	Environmental	Culture and PCR	124	86
Emmanuel et al.^53^	Nigeria	Rectal swab and manure of poultry and cattle	Human and animal	Culture and PCR	167	0
Yilema et al.^29^	Ethiopia	Clinical specimen	Human	Culture	24	10
Solomon et al.^27^	Ethiopia	Indoor air sample	Environmental	Culture	40	3
Katakweba et al.^55^	Tanzania	Faeces of livestock, poultry and human	Animal and Human	Culture and PCR	228	12
Ferede et al.^26^	Ethiopia	Clinical specimen	Human	Culture	15	2
Seid et al.^25^	Ethiopia	Stool sample	Human	Culture	112	7
Joseph et al.^51^	Uganda	Vaginal swab	Human	Culture	49	0
Manamenot et al.^24^	Ethiopia	Stool sample	Human	Culture	220	17
Aziz et al.^47^	Morocco	Cow milk	Animal	Culture and PCR	17	0
Hassan et al.^39^	Egypt	Clinical specimen	Human	PCR	67	17
Houssem et al.^43^	Tunisia	Wild birds’ faeces	Animal	Culture and PCR	79	5
Toru et al.^28^	Ethiopia	Clinical specimen	Human	Culture	22	5
Molechan C et al.^36^	South Africa	Poultry	Animal	Culture and PCR	131	0
Daniel et al.^34^	South Africa	Water	Environmental	Culture and PCR	56	44
Osman et al.^41^	Egypt	Poultry	Animal	Culture and PCR	106	101
Kateete et al.^49^	Uganda	Clinical specimen	Human	Culture	115	20
Frank et al.^37^	South Africa	Faeces, water and soil	Environmental	Culture and PCR	289	176

### Country of origin for the articles

The country in which the articles originated is indicated as follows, eight articles from each country, Ethiopia^22–29^, and South Africa^29–37^, four articles in each country Egypt^38–41^, and Tunisia^42–45^. Another three articles from each of these countries Morocco^46–48^ and Uganda^49–51^ and two articles from each of these countries Nigeria^52,53^, Tanzania^54,55^ and Algeria^56,57^ were included for the study (Table 1).

## Result

### The pooled prevalence of vancomycin resistance *Enterococci*

**Figure 2:**
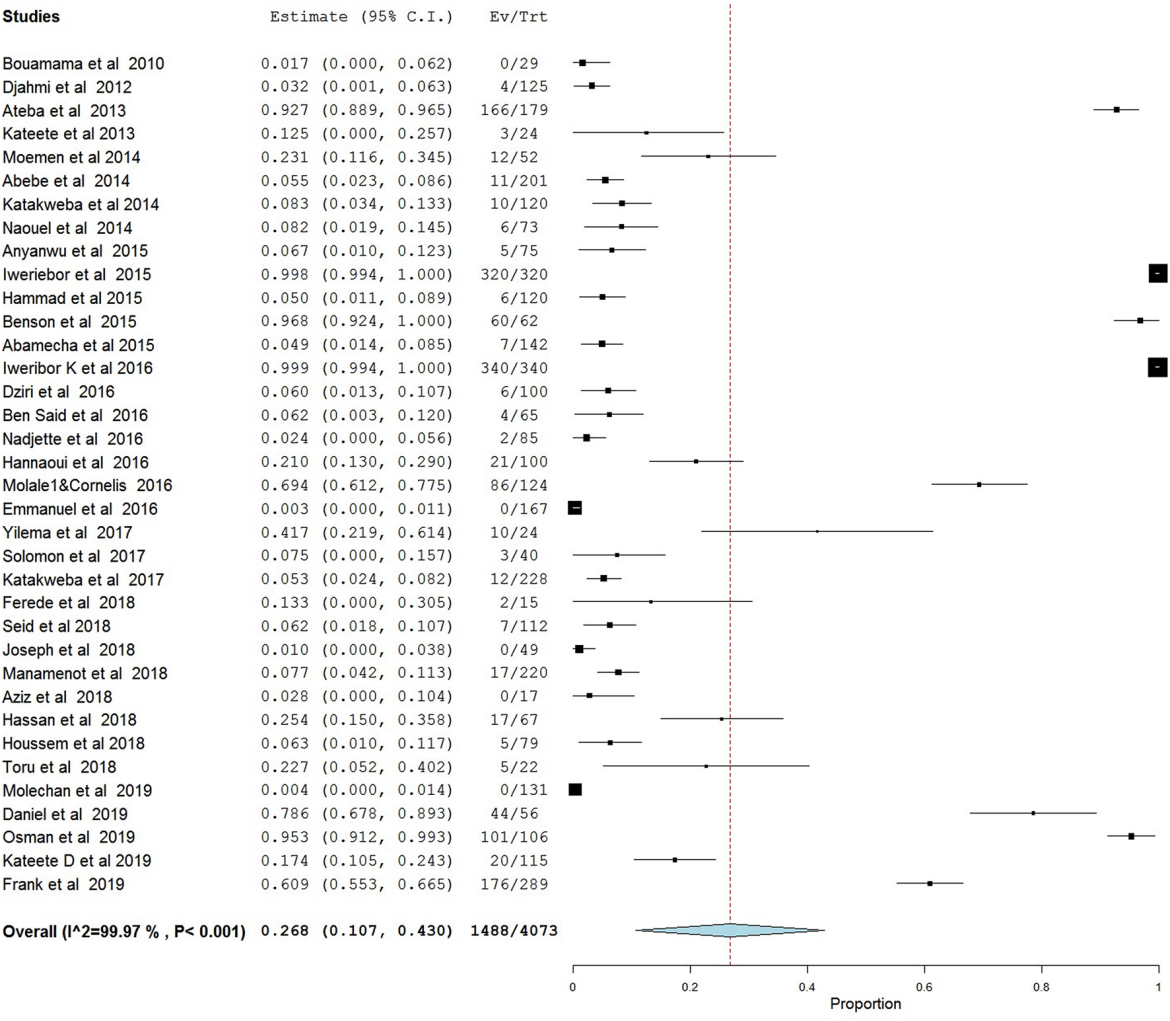
Shows the prevalence of vancomycin-resistant enterococci in Africa in a one-health approach: a systematic review and meta-analysis.

Out of 4073 enterococci isolates described in papers meeting inclusion criteria, 1488 were identified as VRE with an overall pooled prevalence of 26.8% (95% CI; 10.7–43.0, I^2^ = 99.97%; *p* < 0.001) in Africa in a one-health perspective. The meta-analysis indicates that there was considerable heterogeneity among the articles with a consistency index (I^2^) = 99.97% (Fig. 2).

### Sensitivity analysis

**Figure 3:**
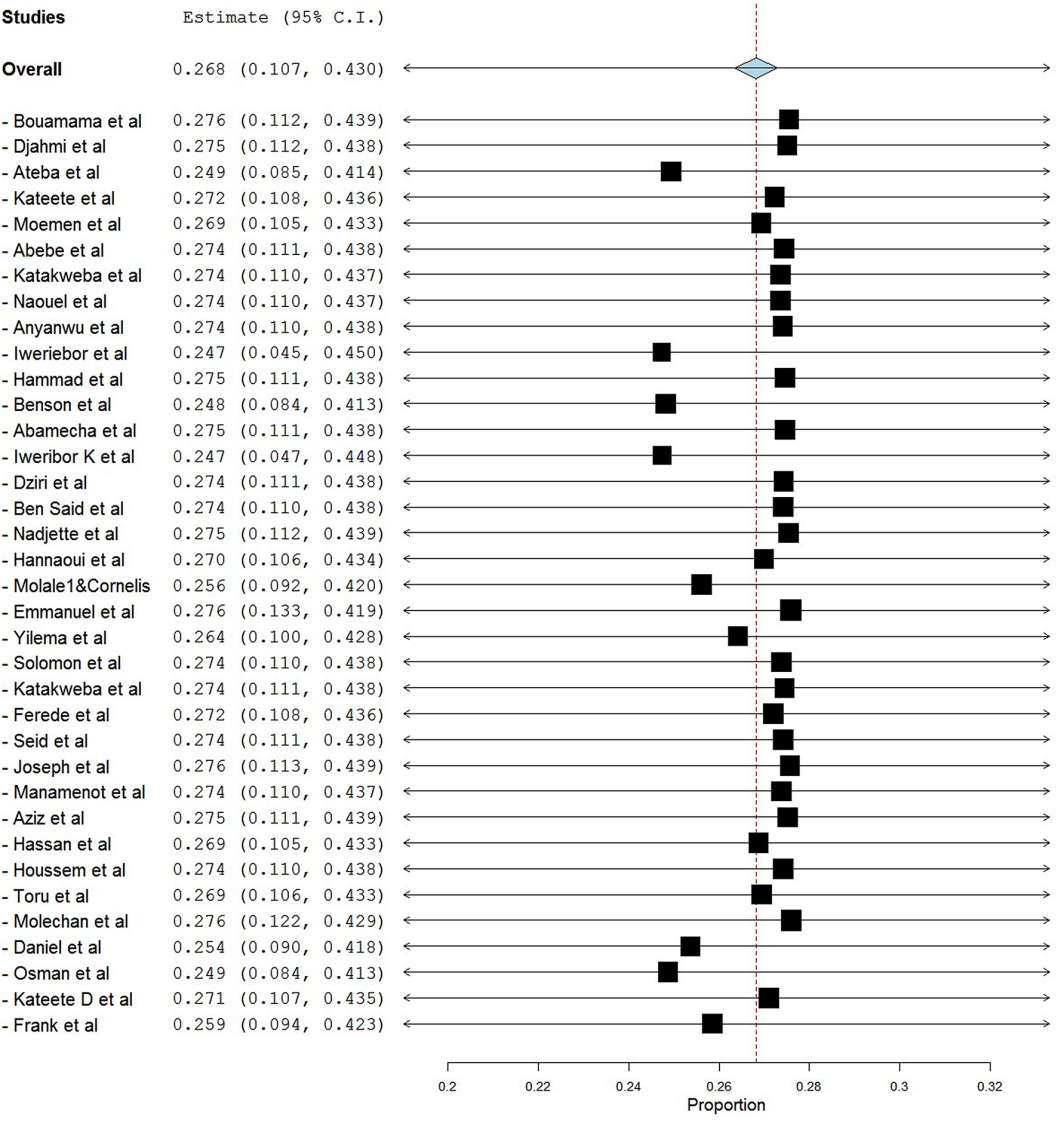
Shows the forest plot for sensitivity analysis of the prevalence of vancomycin-resistant enterococci in Africa in a one-health approach: a systematic review and meta-analysis.

Sensitivity analysis was performed with leave one out analysis showed that there is no difference compared to pooled prevalence 26.8% (95% CI: 10.7–43.0, *p* < 0.001) versus 26.8% (95% CI; 10.7–43.0, *p* < 0.001) (Fig. 3).

### Subgroup analyses

The subgroup analysis performed based on country indicates that the highest prevalence of VRE was in South Africa 74.8% (95% CI; 51–99%; I^2^ = 99.9%; *p* < 0.001) observed followed by, Egypt 37.2% (95% CI; − 17–92%; I^2^ = 99.7%; *p* < 0.001), Uganda 9.8% (95% CI; − 0.027–0.223%; I^2^ = 90.2%; *p* < 0.001), Morocco 8.2% (95% CI =  − 3.0–20.0%; I^2^ = 88.7%; *p* < 0.001), Ethiopia 7.9% (95% CI; 5.0–11.0% ; I^2^ = 60.7%; *p* = 0.01), Tunisia 6.5% (95% CI; 4.0–9.0%, I^2^ = 0; *p* = 0.95), Tanzania 6.1% (95% CI; 3.4–8.8%; I^2^ = 9.27%; *p* < 0.294), Nigeria 2.8% (95% CI; − 3.0–9.0%; I^2^ = 79.1%; *p* = 0.03) and Algeria 2.8% (95% CI; 1.0–5.0%; I^2^ = 0; *p* = 0.71).

Our study conducted a subgroup analysis of VRE based on the laboratory method employed by each study. Accordingly, the laboratory methods grouped into culture and polymerase chain reaction (PCR), PCR only and culture only. It indicates that the highest rates of VRE are from article conducted with PCR 59.2% (95% CI: − 6.8–125.3%; I^2^ = 99.3%; *p* < 0.001), followed by culture and PCR 38.9% (95% CI: 16.1–61.6%; I^2^ = 99.9% *p* < 0.001) and culture only 7.3% (95% CI: 4.8–9.8%; I^2^ = 72.2%; *p* < 0.00).

Our study tried to perform a subgroup analysis of the prevalence of VRE dividing the study publication year into two categories as 2010–2015 and 2016–2019. Accordingly, the prevalence of VRE was higher in the range of 2010–2015 as compared to 2016–2019 (30.3% vs. 25.1%) which is statically significant with *p* < 0.000.

Studies included for our review were from four African regions as defined by African Union commission: South, North, West, and East Africa. No studies were found from countries in the Central Africa Region. The greatest numbers of studies (75%) were from the North an East Africa Regions. Hence, our subgroup analysis indicates that a higher prevalence of VRE was from South African regions 74.8% (95% CI: 51.1–98.5%, I^2^ = 99.9%, *p* < 0.001) followed by, East 17.9% (95% CI: 1.1–34.8%, I^2^ = 99.5%, *p* < 0.001), North 15.9% (95% CI: − 0.6–32.4%, I^2^ = 99.3%, *p* < 0.001) and West 2.8% (95% CI: − 3.3–9.0%, I^2^ = 79.1%, *p* = 0.02). The difference is staticall**y** significant with *p* < 0.000.

Subgroup analysis performed based on the source of sample categorizing as non- human and human source. It indicates that a higher prevalence of VRE detected from environmental sample sources 52.2% (95% CI: 22.5–82.0%, I^2^ = 99.6, *p* < 0.001) followed by animal 30.5% (95% CI: 8.4–52.5%, I^2^ = 99.9%, *p* < 0.001), human 10.2% (95% CI: 6.8–13.7%, I^2^ = 84.5%, *p* < 0.001) and animal and human 3.7% (95% CI: − 1.2–8.6%, I^2^ = 85.2%, *p* < 0.001) (Table 2).

**Table 2:** Subgroup analysis based on laboratory test methods, year of publication, African regions, country and sample source for the pooled prevalence of VRE in Africa in one-health approach.

Subgroups		Studies	The estimated prevalence of VRE (95% CI)	Heterogeneity	
				*p* Val	I^2^ (%)
Country	Ethiopia	8	0.079 (0.046–0.113)	0.013	60.7
	Algeria	2	0.028 (0.006–0.050)	0.710	0
	Egypt	4	0.372 (−0.173–0.917)	< 0.001	99.7
	Morocco	3	0.082 (−0.032–0.196)	< 0.001	88.7
	South Africa	8	0.748 (0.511–0.985)	< 0.001	99.9
	Uganda	3	0.098 (−0.027–0.223)	< 0.001	90.2
	Tunisia	4	0.065 (0.038–0.092)	0.951	0
	Tanzania	2	0.061 (0.034–0.088)	< 0.294	9.27
	Nigeria	2	0.028 (−0.033–0.090)	0.029	79.1
Laboratory methods	Culture and PCR	17	0.389 (0.161–0.616)	< 0.001	99.9
	Culture	17	0.073 (0.048–0.098)	< 0.001	72.2
	PCR	2	0.592 (−0.068–1.253)	< 0.001	99.3
Year of publication	2010–2015	13	0.303 (−0.028–0.634)	< 0.001	99.9
	2016–2019	24	0.251 (−0.002–0.504)	< 0.001	99.9
African regions	South	8	0.748 (0.511–0.985)	< 0.001	99.9
	North	13	0.159 (−0.006–0.324)	< 0.001	99.3
	West	2	0.028 (−0.033–0.090)	0.029	79.1
	East	14	0.078 (0.051–0.106)	< 0.001	72.2
Sample source	Human	14	0.102 (0.068–0.137)	< 0.001	84.5
	Animal	11	0.305 (0.084–0.525)	< 0.001	99.9
	Environmental	8	0.522 (0.225–0.820)	< 0.001	99.6
	Human and animal	3	0.037 (−0.012–0.086)	0.001	85.2
	Overall	36	0.268 (0.107–0.430)	< 0.001	99.9

## Discussions

Vancomycin is one of a limited number of antibiotics that can be used to treat infections in humans resulting from Gram-positive multidrug-resistant organisms (MDRO) including *Enterococci*^58^. In the late 1980s, the emergence of VRE in European hospitals followed by isolation from Danish raw minced pork and frozen poultry generated global concern^59^. One Health is the concept that the optimum health for people, animals, and the environment should all be considered through the ongoing cooperative efforts of scientists and practitioners in a variety of disciplines^60^.

Our study based on the data available from studies in Africa on VRE in which animal, human, and environmental sources of samples had been specified were analyzed to determine the pooled prevalence of VRE. The overall prevalence of VRE was (26.8%) in Africa from different sample sources. This prevalence is higher than reported in the studies conducted in Iraq (14%)^61^, Europe (2.7%)^1^, (13%)^62^, Thailand (10.3%)^63^, South America (6%)^64^. However, it is comparable to a study from Latin America (30%)^65^. These differences may be due to the source of the sample we used for the analysis is from different sources and may be due to the enterococcus population structure selected overtime which is highly resistant for environmental conditions and different antibiotics^2,66^.

The subgroup analysis at the country level showed that there is a pronounced difference of VRE in different countries, which ranged from (74.8%) in South Africa to (2.8%) in Algeria and Nigeria which is statically significant with *p* < 0.000. This variation might be due to sampling source difference, sample size, laboratory method used, year of publication, and the number of studies included for the meta-analysis.

Our study also performed a subgroup meta-analysis based on the laboratory method used for isolation and identification of VRE. It showed that the higher the technique engaged by the studies for identification of VRE, the more sensitive and specific for detection of VRE in which studies conducted with PCR primers for isolation of higher prevalence of VRE, whereas those conducted with conventional culture were less likely to detect VRE. Some studies reported in a comparison of PCR and culture supports that the former technique is more sensitive and specific than later one for the identification of VRE^67–69^.

Our study revealed that a reduction of VRE from (2010–2015) to (2016–2019) (30.3%) versus (25.1%). In contrast to our finding a study from Europe bared increment in VRE from 2012 to 2018 which was (8.1%) to (19.1%)^62^, in Brazil from 2006 to 2009 from (2.5%) to (15.5%)^70^. The disagreement might be due to study period variance, the area covered, sample type used, the ability of detection of laboratories dissimilarity.

Analysis of VRE in African regions showed that there was a high prevalence in the South African region (74.8%) almost twenty-six times of West Africa and four times than of North and East African regions. The difference can be explained it might be due to the laboratory method used for detection and identification of VRE^67,69^, the sample difference^71^ and overall antibiotics usage in human^72,73^ and animal^74–76^.

The sample source in which we categorized in human, animal and environmental sources for the sake of subgroup meta-analysis showed that a higher prevalence of VRE was isolated from environmental, followed by the animal source as compared to a human source. This may be due to most of the articles included based on our inclusion criteria is from non-human sources as different wild and domestic animal wastes or by-products, poultry, birds, and the environmental sample was compiled for analysis. The other reason is probably due to the intensive conditions in which these animals maintained for different antibiotics as a kind of growth promoter^77,78^. This part of the study strained the one health approach, which is an important way of combating antibiotics resistance that worsens the world; now a day’s^77,79^.

### Strength and limitation of the study

The strength of our review and meta-analysis is, it presented an all-inclusive data about VRE in Africa. It offered a subgroup analysis of data based on country, laboratory method used, African regions, year of publication, and source of the sample. Even if we included the most common databases for searching, our data has a limitation of addressing all search engines. It also did not identify which species of enterococci with resistances are commonly reported.

## Conclusion

Our meta-analysis finding demonstrated that there is a high prevalence of VRE circulating in Africa. The subgroup analysis indicates that a high prevalence of VRE isolated from South African region. Similarly, studies conducted with PCR laboratory method isolated the highest VRE. Additionally, our study showed that the prevalence decreasing over time. Environmental sample source is with a higher VRE as compared to the human and animal sample source. Thus, a means of prevention and control targeting humans, animals, and environments based on regional, and country perspectives should be practised in the continent to alleviate the infection with VRE.

## Supplementary information

Supplementary information.

## Data Availability

All the data supporting the findings can be obtained from the corresponding author.
