# Supplementary material for: Prevalence of vancomycin-resistant enterococcus in Africa in one health approach: a systematic review and meta-analysis
Source: Sci Rep. 2020 Nov 25;10:20542. doi: 10.1038/s41598-020-77696-6 (PMC7688635; doi:10.1038/s41598-020-77696-6)
Supplement: Supplementary file 1 — Supplementary information. [file 41598_2020_77696_MOESM1_ESM.pdf]

Prevalence of Vancomycin Resistant Enterococcus in Africa in One Health Approach: A Systematic Review and Meta-analysis

Authors: Tsegaye Alemayehu*, Mengistu Hailemariam

Affiliation: [1]Hawassa University College of Medicine and Health Sciences, School of Medical Laboratory Sciences

Mailing Address:

TA: alemayehutsegaye@ymail.com

MH: mengamariam@yahoo.com

The Quality of articles for the study of the prevalence of VRE in Africa in one health approach: a systematic review and meta-analysis. Based on National heart, lung and blood institute check list 1- Yes, 0-No

Authors	NHL, 14-point Checklist														Total score	Grade
	1	2	3	4	5	6	7	8	9	10	11	12	13	14		
Molechan C. et al	1	1	NR	1	0	0	0	NA	NA	NA	1	NA	NA	0	4/8	Poor
Daniel P. et al	1	1	NA	1	0	0	0	NA	NA	NA	1	NA	NA	0	5/8	Good
Osman K. et al	1	1	NR	1	0	0	0	NA	NA	NA	1	NA	NA	0	4/8	Poor
Bouamama L. et al	1	1	NR	1	0	0	0	NA	NA	NA	1	NA	NA	0	4/8	Poor
Iweriebor B. et al	1	1	NR	1	1	0	0	NA	1	NA	1	NA	NA	0	6/9	Good
Ateba C. et al	1	1	NR	1	1	0	0	NA	0	NA	1	NA	NA	0	5/9	Fair
Moemen D. et al	1	1	NR	1	1	0	0	NA	1	NA	1	NA	NA	1	7/9	Good
Dziri R. et al	1	1	NR	1	1	0	0	NA	1	NA	1	NA	NA	0	6/9	Good
Anyanwu M. et al	1	1	NR	1	1	0	0	NA	0	NA	1	NA	NA	0	5/9	Fair
Iweriebor B. et al	1	1	NR	1	1	0	0	NA	0	NA	1	NA	NA	0	5/9	Fair
Yilema A. et al	1	1	1	1	1	0	0	NA	1	NA	1	NA	NA	1	8/10	Good
Kateete D. et al	1	1	NR	1	1	0	0	NA	0	NA	1	NA	NA	0	5/9	Fair
Solomon F. et al	1	1	NR	NA	1	0	0	NA	0	NA	1	NA	NA	0	4/8	Poor
Ferede Z. et al	1	1	1	1	1	0	0	NA	1	NA	1	NA	NA	1	8/10	Good
Seid A. et al	1	1	1	1	1	0	0	NA	1	NA	1	NA	NA	1	8/10	Good
Joseph N. et al	1	1	NR	1	1	0	0	NA	1	NA	1	NA	NA	1	8/9	Good
Manamenot A et al	1	1	1	1	1	0	0	NA	1	NA	1	NA	NA	1	8/10	Good
Abebe W. et al	1	1	1	0	1	0	0	NA	1	NA	1	NA	NA	1	7/10	Good
Ben Said et al	1	1	NA	1	1	0	0	NA	0	NA	1	NA	NA	0	5/9	Fair
Frank F. et al	1	1	NA	1	1	0	0	NA	1	NA	1	NA	NA	0	7/10	Good
Nadjette B. et al	1	1	1	1	1	0	0	NA	0	NA	1	NA	NA	0	7/10	Good
Djahmi N. et al	1	1	NR	1	1	0	0	NA	1	NA	1	NA	NA	1	7/9	Good
Aziz B. et al	1	1	NA	1	1	0	0	NA	0	NA	1	NA	Na	0	4/9	Poor
Hammad A. et al	1	1	NA	1	1	0	0	NA	0	NA	1	NA	NA	0	5/9	Fair
Toru M. et al	1	1	1	1	1	0	0	NA	1	NA	1	NA	NA	0	7/10	Good
Hannaoui I. et al	1	1	NR	1	0	0	0	NA	0	NA	1	NA	NA	0	4/9	Poor
Hassan R. et al	1	1	NR	1	0	0	0	NA	0	NA	1	NA	NA	0	4/9	Poor
Iweriebor B. et al	1	1	NR	1	0	0	0	NA	0	NA	1	NA	NA	0	4/9	Poor
Katakweba A. et al	1	1	NR	1	0	0	0	NA	1	NA	1	NA	NA	1	6/9	Good
Kateete D. et al	1	1	NR	0	0	0	0	NA	1	NA	1	NA	NA	0	4/9	Poor
Katakweba E. et al	1	1	NR	0	1	0	0	NA	1	NA	1	NA	NA	0	5/9	Fair
Naouel K. et al	1	1	NR	1	1	0	0	NA	0	NA	1	NA	NA	0	5/9	Fair
Molale1&Cornelis	1	1	NR	1	1	0	0	NA	0	NA	1	NA	NA	0	5/9	Fair
Emmanuel O. et al	1	1	NR	0	0	0	0	NA	0	NA	1	NA	NA	0	3/9	Poor
Abamecha A. et al	1	1	NR	1	1	0	0	NA	1	NA	1	NA	NA	0	6/9	Good
Houssem B. et al	1	1	NR	1	0	0	0	NA	0	Na	1	NA	NA	0	4/9	Poor

Key: NR –not reported, NA- not applicable, NHL – National heart, lung and blood institute
